# Recent Achievements of Epicardial Patch Electronics Using Adhesive and Conductive Hydrogels

**DOI:** 10.3390/gels11070530

**Published:** 2025-07-09

**Authors:** Su Hyeon Lee, Jong Won Lee, Daehyeon Kim, Gi Doo Cha, Sung-Hyuk Sunwoo

**Affiliations:** 1 Department of Chemical Engineering, Kumoh National Institute of Technology, Gumi 39177, Republic of Korea; soohyun9803@kumoh.ac.kr (S.H.L.); eogus9462@naver.com (D.K.); 2 Department of Systems Biotechnology, Chung-Ang University, Anseong 17546, Republic of Korea; ljw0971@cau.ac.kr

**Keywords:** epicardial electronics, soft electronics, biomaterials, adhesive hydrogels, conductive hydrogels

## Abstract

Implantable cardiac devices are critical in improving patients’ quality of life through precise and continuous interaction between the device and pathological cardiac tissue. Due to the inherently rigid nature of conventional devices, several complications arise when interacting with soft cardiac tissue, caused by a mechanical mismatch between the device and myocardium. This leads to the excessive formation of fibrous tissue around the implanted device, ultimately compromising both device functionality and tissue health. To address these challenges, flexible electronics based on polymers and elastomers significantly softer than conventional rigid metals and silicon have been explored. The epicardial approach enables the device to conform to the curved myocardial surface and deform synchronously with cardiac motion, thereby improving mechanical compatibility. However, modulus mismatches between soft polymers and cardiac tissue can still lead to mechanical instability and non-uniform adhesion, potentially affecting long-term performance. This review comprehensively summarizes recent research advancements in epicardial patch electronics based on bioadhesive and conductive hydrogels. We emphasize current research directions, highlighting the potential of hydrogels in epicardial electronics applications. Critical discussion includes recent trends, ongoing challenges, and emerging strategies aimed at improving the properties of hydrogel-based epicardial patches. Future research directions to facilitate clinical translation are also outlined.

## 1. Introduction

Recent advances in implantable bioelectronics have significantly enhanced the precision and continuity of real-time monitoring and on-demand stimulation in biomedical applications [1]. Among various fields, cardiovascular medicine has particularly benefited from these developments, as precise and continuous interaction between bioelectronic devices and pathological cardiac tissue is not only crucial for improving patients’ quality of life but also essential in life-threatening situations [2]. Implantable cardiac devices have long served as the frontline defense against chronic cardiovascular diseases; however, their inherently rigid nature has introduced several complications when interfacing with soft cardiac tissue [3]. The primary issue arises from the mechanical mismatch between the rigid device and the dynamically moving myocardium, leading to adverse biological responses [4,5]. While acute inflammatory reactions are inevitable for all implanted materials, prolonged and non-compliant interactions between rigid electronics and soft tissues exacerbate both acute and chronic inflammatory responses. The fibrotic tissue around the implanted device can be formed due to inflammatory responses, and it might ultimately compromise both device functionality and tissue health [6].

To address these challenges, flexible electronics based on polymers such as PDMS, polyurethane (PU), polyimide (PI), poly(N-isopropylacrylamide) (PNIPAm), and poly(2-hydroxyethyl methacrylate) (PHEMA) have been explored as softer alternatives to rigid metals and silicon in cardiac bioelectronics [7,8]. These flexible electronic systems have facilitated an epicardial approach, wherein bioelectronic patches interact with the outer layer of the myocardium (epicardium) rather than the inner layer (endocardium), as seen in traditional intravenous or endocardial devices [9]. This epicardial approach enables the device to conform to the myocardial surface and deform synchronously with cardiac motion, thereby improving mechanical compatibility [10]. Compared to conventional endocardial implants, epicardial patches offer the advantage of spatially resolved electrophysiological monitoring across the entire heart surface and precise, localized electrical stimulation for therapeutic interventions.

**Figure 1:**
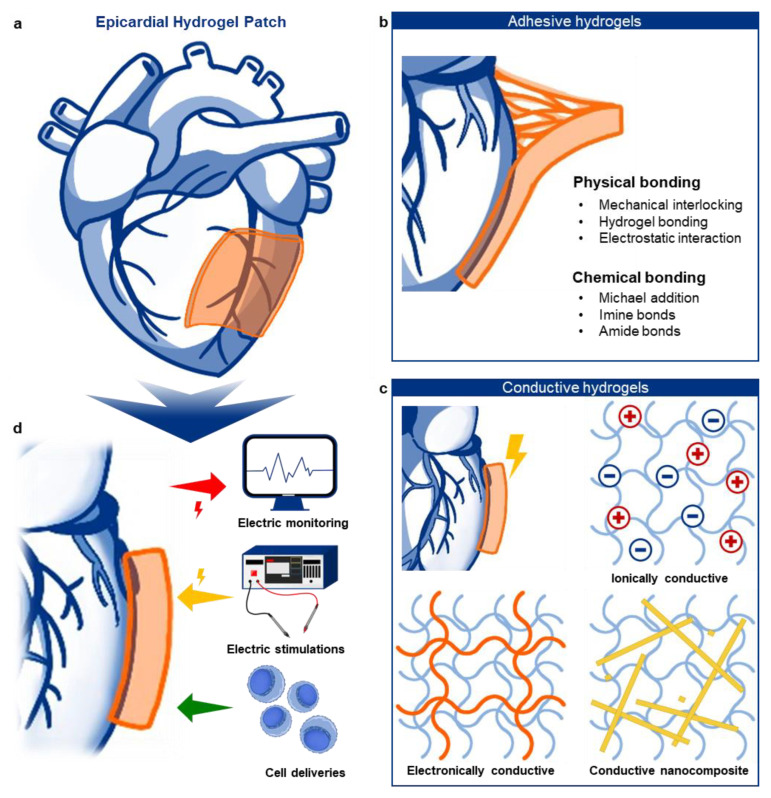
Schematic illustrations describing overall flow of the review. (**a**) Epicardial hydrogel patch placed on the heart surface for cardiac interfacing. (**b**) Bioadhesive hydrogels enabling strong tissue adhesion via physical and chemical mechanisms. (**c**) Conductive hydrogels classified by their conduction mechanisms: ionic (top right), conductive polymer-based (bottom left), and nanomaterial-based (bottom right). (**d**) Applications of multifunctional hydrogels for epicardial monitoring, electrical stimulation, and therapeutic intervention.

Despite these advances, flexible polymer-based electronics still face limitations in achieving fully conformal contact with the constantly moving myocardium. For example, the modulus of myocardial tissue typically ranges from 10 to 100 kPa. Instead, soft polymers such as PDMS (~100 kPa–1 MPa), polyurethane (PU) (~100 kPa–10 MPa), polyimide (PI) (~1–3 GPa), poly(N-isopropylacrylamide) (PNIPAm) (~10–100 kPa), and poly(2-hydroxyethyl methacrylate) (PHEMA) (~10–500 kPa) have been explored to match the mechanical compliance required for cardiac bioelectronics. Still, the modulus mismatch between even soft polymers and cardiac tissue still exists, which can lead to mechanical instability and non-uniform adhesion, potentially affecting long-term performance [11]. To overcome this issue, intrinsically soft and tissue-like materials such as hydrogels have gained significant attention as next-generation materials for cardiac bioelectronics (Figure 1a) [12]. Hydrogels, with their ultrasoft mechanical properties, excellent biocompatibility, bioadhesiveness, and inherent conductivity, present a promising platform for epicardial patches [13,14]. In this mini review, we will introduce recently reported bioadhesive and conductive hydrogels with a brief discussion of their fundamental mechanisms (Figure 1b,c). We will then examine recent progress in applying these advanced hydrogels for epicardial monitoring, electrical stimulation, and therapeutic interventions (Figure 1d). Finally, we will highlight the current challenges and future prospects in this emerging field.

## 2. Adhesive Hydrogel

In physiological environments, where tissues remain continuously hydrated, achieving strong adhesion under wet conditions is crucial to maintaining a seamless tissue-hydrogel interface [15,16,17,18]. Robust adhesion is particularly important for ensuring mechanical stability and preventing detachment under the dynamic motions of organs, especially the heart [19,20]. However, conventional hydrogels often exhibit weak adhesion due to the presence of a hydration layer at the interface, which impairs direct bonding with tissue surfaces [15,19]. To address this challenge, various strategies have been developed to enhance tissue–hydrogel interfacial adhesion, employing both physical and chemical bonding interactions [15,21,22,23]. This section discusses key strategies for improving hydrogel adhesion, categorized into physically adhesive hydrogels and chemically adhesive hydrogels.

### 2.1. Physically Adhesive Hydrogel

Physical adhesion mechanisms rely on non-covalent interactions between hydrogel and tissue, which enable reversible yet effective attachment [21,23].

**Figure 2:**
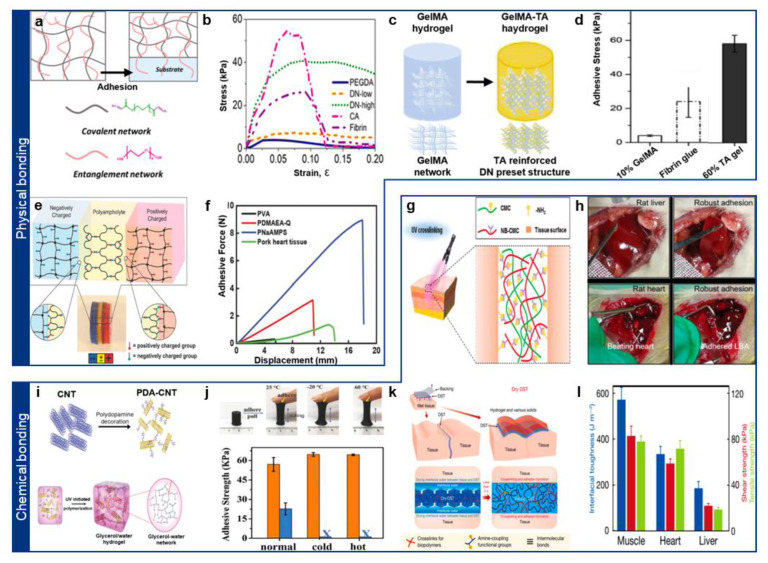
(**a**) Schematic illustration of the entanglement-based adhesive hydrogel. Reprinted with permission [24]. Copyright 2019 © American chemical Society. (**b**) Lap shear tests showing the adhesion performance of the hydrogels on glass slides. Reprinted with permission [24]. Copyright 2019 © American chemical Society. (**c**) Material design of the hydrogen bond-based GelMA-TA adhesive hydrogel. Reprinted with permission [34]. Copyright 2018 © Elsevier Inc. (**d**) Demonstration of the superior adhesion strength of GelMA/TA gel compared to fibrin glue and GelMA gel. Reprinted with permission [34]. Copyright 2018 © Elsevier Inc. (**e**) Schematic illustration of the polyampholyte (PA)-based hydrogel adhesion mechanism. Reprinted with permission [35]. Copyright 2015 © John Wiley & Sons. (**f**) Adhesive force of PA-based hydrogel on various substrates measured by lab shear test. Reprinted with permission [35]. Copyright 2015 © John Wiley & Sons. (**g**) Schematic illustration of the Carboxymethyl chitosan (CMC)-based hydrogel adhesion mechanism. Reprinted with permission [36]. Copyright 2020 © John Wiley & Sons. (**h**) Demonstration of the fast and strong adhesion in rat liver (top) and beating heart (bottom). Reprinted with permission [36]. Copyright 2020 © John Wiley & Sons. (**i**) Adhesive force of Material design of the polydopamine (PDA)-decorated carbon nanotubes (CNTs)-based adhesive hydrogel. Reprinted with permission [37]. Copyright 2017 © John Wiley & Sons. (**j**) Optical image of stable adhesion at harsh temperatures (top) and the corresponding adhesive strength data in the graph (bottom). Reprinted with permission [37]. Copyright 2017 © John Wiley & Sons. (**k**) Schematic illustration of the dry crosslinking adhesion mechanism of the dry double-sided tape (DST). Reprinted with permission [38]. Copyright 2019 © Springer Nature. (**l**) Interfacial toughness and shear strength between porcine skin and heart adhered by DST. Reprinted with permission [38]. Copyright 2019 © Springer Nature.

One widely employed mechanism is polymer entanglement, where polymer chains from the hydrogel physically intertwine with those of the tissue, forming a mechanically interlocked structure that enhances adhesion (Figure 2a) [24]. Polymer chains begin to significantly entangle with one another—thereby increasing the viscosity of a melt or solution—only when their molecular weight exceeds a critical threshold. This leads to the high entanglement between the hydrogel polymer and tissue polymer, resulting in the adhesive hydrogel–tissue interface [25,26]. This entanglement also facilitates energy dissipation through non-covalent interactions, improving adhesion stability [24,27]. For example, Chen et al. utilized hydrophilic and biocompatible polyethylene glycol (PEG), which readily intertwines with tissue surface polymers, thereby ensuring stable adhesion under wet conditions (Figure 2b) [24]. This approach has gained attention as a biocompatible strategy, as it avoids cytotoxic chemical crosslinkers [24,28,29].

Hydrogen bonding represents another key physical interaction for hydrogel adhesion. Tissue surfaces contain abundant functional groups such as hydroxyl (-OH), carboxyl (-COOH), and primary amines (-NH2), which can form hydrogen bonds with complementary groups in hydrogels [23,30,31]. Since hydrogen bonds are relatively strong among non-covalent interactions, multiple hydrogen bonds can collectively yield adhesion strengths comparable to chemical bonding [21,32]. Representative hydrogen-bonding moieties include catechol and polyphenols, which exhibit a strong affinity toward tissue surfaces [32,33]. For instance, B. Liu et al. developed a highly adhesive hydrogel incorporating tannic acid (TA), a polyphenol, which facilitated strong adhesion by forming multiple hydrogen bonds between gelatin methacrylate (GelMA) and tissue functional groups (Figure 2c,d) [34].

However, hydrogen bonding is inherently sensitive to water, as competing interactions with surrounding water molecules can weaken adhesion [21,39]. To overcome this limitation, researchers have explored electrostatic interactions, which involve charged functional groups naturally present in biological tissues under physiological conditions [39]. Carboxylate (-COO^−^) and ammonium (-NH_3_^+^) ions contribute to electrostatic adhesion, as seen in hydrogels composed of alginate and chitosan—two biopolymers capable of engaging in electrostatic interactions with tissue surfaces. However, single-charged polymers often exhibit relatively weak adhesion and may induce cytotoxic effects due to charge imbalances [40,41]. To mitigate this, polyampholyte hydrogels, which contain both positive and negative charges, have been developed to enhance adhesion stability (Figure 2e) [35,42]. For example, neutral polyampholytes synthesized by copolymerizing sodium 4-vinylbenzene/sulfonate and [2-(acryloyloxy)ethyl]trimethylammonium chloride demonstrated strong and rapid adhesion to both positively and negatively charged tissue surfaces, highlighting their potential for biomedical applications (Figure 2f) [35].

### 2.2. Chemically Adhesive Hydrogel

While physically adhesive hydrogels offer reversible adhesion, their bond strength is often insufficient for long-term applications [21]. To address this, chemically adhesive hydrogels have been developed, forming stable covalent bonds with tissue biomolecules [39,43].

Tissue surfaces are rich in functional groups derived from amino acids, such as primary amines (-NH_2_), carboxyl (-COOH), imidazole (-C_3_H_4_N_2_), and thiol (-SH) groups [39,44,45]. These functional groups serve as anchoring sites for hydrogel adhesion. Cyanoacrylates, widely known synthetic adhesives, undergo anionic polymerization, forming strong bonds via Michael addition between amine groups in tissues and vinyl groups in the adhesive [46,47]. Although cyanoacrylate-based adhesives provide rapid adhesion, their clinical use is limited due to toxic byproducts and water-initiated degradation [23,47].

To achieve strong adhesion while ensuring biocompatibility, aldehyde-functionalized polymers have gained attention [20,23,48]. Aldehydes exhibit relatively low reactivity with water and readily form imine bonds via Schiff base reactions with primary amines on tissue surfaces (Figure 2g) [36,49]. These imine bonds enable rapid and robust adhesion, as demonstrated by hydrogel patches exhibiting high adhesion strength on rat liver and beating heart models in vivo (Figure 2h) [36].

Recent efforts have focused on combining physical and chemical interactions to further enhance adhesion strength [21,46]. Inspired by mussel adhesion mechanisms, researchers have utilized dopamine-functionalized hydrogels, where dopamine catechol groups form strong hydrogen bonds with tissue surfaces while simultaneously undergoing oxidation-mediated covalent bonding via Michael addition (Figure 2i) [37]. Recent studies have shown that glycerol enhances catechol adhesion stability under both low and high temperatures, expanding its biomedical applicability (Figure 2j) [37].

Another widely used chemical adhesion strategy involves N-hydroxysuccinimide (NHS) chemistry [49]. NHS esters react with primary amines in tissue proteins, forming amide bonds that ensure stable adhesion [49,50]. NHS-functionalized polymers have been broadly utilized for in situ hydrogel adhesion (Figure 2k) [38,50]. Expanding on this concept, X. Zhao’s group introduced hygroscopic and anisotropic swelling mechanisms to actively remove surface moisture, thereby enhancing adhesion via both NHS-primary amine bonding and additional physical interactions [38]. This approach demonstrates the potential for universal hydrogel-tissue adhesion (Figure 2l) [38].

## 3. Conductive Hydrogel

With the rapid advancements in soft bioelectronics, there has been increasing interest in utilizing hydrogels as active electronic components [15,51,52,53]. Unlike traditional rigid electronic materials, hydrogels offer exceptional biocompatibility, mechanical compliance, and water-retentive properties, making them well-suited for biomedical applications [15,54,55]. However, pristine hydrogels exhibit inherently low electrical conductivity, limiting their direct application in bioelectronic devices [56,57]. To overcome this limitation, various strategies have been developed to enhance the electrical conductivity of hydrogels through the integration of ionic or electronic charge carriers [56,58]. This section explores different approaches for imparting conductivity to hydrogels, categorized into three main strategies: (i) ionically conductive hydrogels, which rely on mobile ions for charge transport and are simple and cost-effective, though they often suffer from ion leakage and limited long-term stability; (ii) conductive polymer-based hydrogels, which leverage conjugated polymer networks for electronic conductivity but may exhibit mechanical rigidity and require complex synthesis that increase fabrication complexity and cost; and (iii) nanocomposite conductive hydrogels, which incorporate metallic or carbon-based nanomaterials to achieve synergistic improvements in conductivity and softness, albeit with higher fabrication costs and potential concerns regarding long-term biocompatibility.

### 3.1. Ionically Conductive Hydrogel

**Figure 3:**
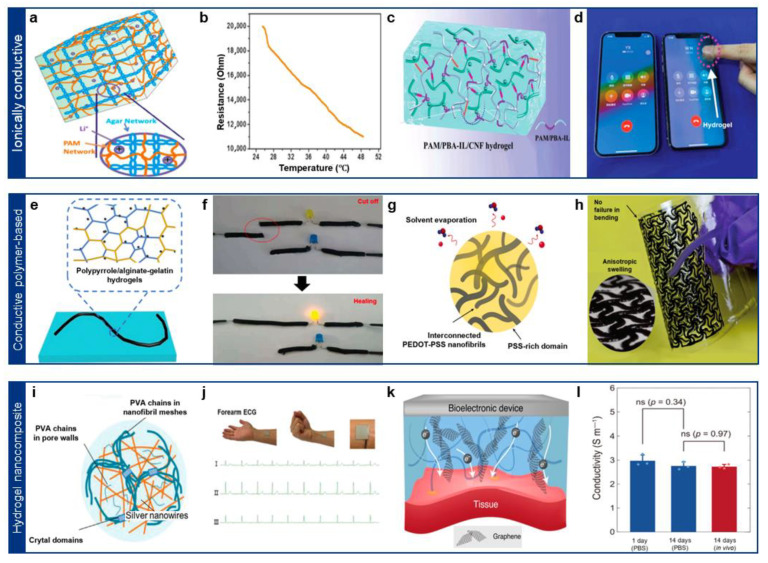
(**a**) Schematic illustration of the electrolyte conductive hydrogel. Reprinted with permission [59]. Copyright 2019 © American chemical Society. (**b**) Reduction in hydrogel resistance due to increased ion mobility with rising temperature. Reprinted with permission [59]. Copyright 2019 © American chemical Society. (**c**) Schematic illustration of the liquid ion-based conductive hydrogel. Reprinted with permission [66]. Copyright 2022 © John Wiley & Sons. (**d**) Demonstration of e-skin for smartphone touch interface. (**e**) Schematic illustration of the PPy-based conductive hydrogel. Reprinted with permission [66]. Copyright 2019 © Royal Society of Chemistry. (**f**) Demonstration of the self-healing conductivity of hydrogel. Reprinted with permission [73]. Copyright 2019 © Royal Society of Chemistry. (**g**) Material design and synthetic method of the Pure PEDOT:PSS conductive hydrogel. Reprinted with permission [74]. Copyright 2019 © Springer Nature. (**h**) Patterning of pure PEDOT:PSS hydrogel and demonstration of their flexibility. Reprinted with permission [74]. Copyright 2019 © Springer Nature. (**i**) Schematic illustration of the AgNWs-based conductive hydrogel. Reprinted with permission [75]. Copyright 2023 © John Wiley & Sons. (**j**) Demonstration of the hydrogel ECG signal sensor on the forearm, capable of sensing even during dynamic movements. Reprinted with permission [75]. Copyright 2023 © John Wiley & Sons. (**k**) Material design of the graphene nanocomposite-based e-bioadhesive interface. Reprinted with permission [19]. Copyright 2020 © Springer Nature. (**l**) Demonstration of the long-term stable conductivity of the hydrogel interface. Reprinted with permission [19]. Copyright 2020 © Springer Nature.

One of the simplest and most effective methods to confer conductivity to hydrogels is by incorporating strong electrolytes, such as sodium chloride (NaCl) and lithium chloride (LiCl), into the hydrogel network [59,60,61]. These electrolytes serve as mobile ion carriers, allowing charge transport within the hydrated matrix. For instance, Yang et al. developed a LiCl-integrated agar/polyacrylamide (PAAm) hydrogel, which exhibited enhanced electrical conductivity at high temperatures due to the temperature-dependent mobility of Li^+^ ions (Figure 3a) [59]. The significant variation in ionic mobility with temperature suggests potential applications in temperature-sensitive bioelectronic sensors (Figure 3b) [59].

However, a major challenge associated with electrolyte-based hydrogels is ion leakage into surrounding biological fluids, which can degrade long-term performance and disrupt local ion homeostasis [62,63]. To mitigate this issue, researchers have explored ionic liquids (ILs)—organic salts that remain in a liquid state at room temperature [64]. ILs exhibit high ionic conductivity, low volatility, and strong electrostatic interactions, making them ideal candidates for stable, leakage-resistant conductive hydrogels [64,65]. Yao et al. introduced a phenylboronic acid-ionic liquid (PBA-IL)/cellulose nanofibril (CNF)/acrylamide (AM) hydrogel, which leveraged ILs to enhance ionic conductivity while forming stable conductive pathways via interactions between negatively charged CNFs and ILs (Figure 3c,d) [66]. These advancements suggest promising applications for long-term implantable bioelectronics requiring stable ionic transport.

### 3.2. Conductive Polymer-Based Hydrogel

Conductive polymers (CPs) are another widely explored approach for imparting electronic conductivity to hydrogels [56,61,67]. These polymers, characterized by conjugated π-electron systems in their backbone, enable efficient electron transport [68]. Common conductive polymers used in hydrogel-based bioelectronics include poly(3,4-ethylenedioxythiophene) polystyrene sulfonate (PEDOT:PSS), polypyrrole (PPy), and polyaniline (PANi) [68,69,70,71]. PEDOT:PSS is favored for its water-based processability, solvent-doped mixed high conductivity, and proven cytocompatibility [72]. PPy offers high charge-storage and facile in situ electropolymerization but forms brittle, oxidation-prone networks. PANi supplies pH-switchable redox activity, yet its conductivity and stability diminish near neutral pH owing to acid dopants.

While these materials exhibit high conductivity, their intrinsic rigidity and hydrophobic nature limit their seamless integration with hydrogel matrices [67,73]. To address this, recent studies have focused on hybridizing conductive polymers with hydrophilic, mechanically compliant networks [15,73]. For instance, PPy was incorporated into a flexible alginate/gelatin hydrogel, creating a biocompatible conductive matrix (Figure 3e) [73]. Additionally, the dynamic bonds within the alginate/gelatin network conferred self-healing properties, making the hydrogel highly suitable for smart bioelectronic sensors and wearable devices (Figure 3f) [73].

Incorporating conducting polymers into hydrogels enhances their electrical conductivity; however, due to the intrinsic rigidity of conjugated π-electron backbones in most conducting polymers, this often leads to reduced mechanical flexibility. These stiff backbones limit the chain mobility and deformability of the hydrogel network, reflecting a fundamental trade-off between electrical performance and mechanical compliance in conductive hydrogel systems [15,74,76]. To address this, Zhao’s group developed a pure PEDOT:PSS nanofibril-based hydrogel with improved conductivity and mechanical properties [74]. Using a dry-annealing technique, which involved adding a volatile solvent to the PEDOT:PSS solution, they achieved a highly conductive and stretchable hydrogel (Figure 3g) [74]. The resulting material exhibited exceptional mechanical stability in wet physiological environments, making it particularly promising for cardiac soft bioelectronics (Figure 3h) [74].

### 3.3. Conductive Hydrogel Nanocomposite

A third approach to enhancing hydrogel conductivity involves integrating conductive nanomaterials, such as metallic and carbon-based nanomaterials, into the hydrogel network [15,55,62]. This strategy allows synergistic enhancement of electrical conductivity while preserving the hydrogel’s intrinsic softness and flexibility [15,55,62].

Among metallic nanomaterials, silver nanowires (Ag NWs) have been widely used as 1D conductive fillers to form percolation networks within hydrogels [75,77]. These networks facilitate efficient charge transport, enabling high electrical conductivity without significantly compromising mechanical properties (Figure 3i) [75]. For example, Ag NW/PVA-nanofibril-based hydrogels exhibited improved mechanical robustness and electrical conductivity, making them highly suitable for wearable bioelectronic patches capable of detecting abnormal biosignals (Figure 3j) [75].

Similarly, graphene oxide (GO), a 2D carbon nanomaterial, has been extensively studied for its tunable electrical and mechanical properties [19,78,79]. The degree of GO reduction plays a crucial role in determining the hydrogel’s performance: higher reduction levels improve electrical conductivity but often degrade mechanical flexibility (Figure 3k) [19,55]. GO/PVA-based hydrogels have been reported as biocompatible electrical bioadhesive interfaces, demonstrating stable electrical signal transmission between biological tissues and electronic devices [19]. Notably, these hydrogels retained their electrical and mechanical integrity even after two weeks of immersion in physiological fluids, highlighting their long-term stability for bioelectronic applications (Figure 3l) [19]. Beyond these representative cases, a broader range of conductive nanomaterials—including liquid metals, MXenes, carbon nanotubes, and carbon black—have also been employed to tailor the electro-mechanical properties of hydrogels. Table 1 summarizes these materials, their conductivity enhancement mechanisms, and their respective benefits and trade-offs for bioelectronic applications.

**Table 1:** Classification and characteristics of nanomaterials employed to enhance the conductivity of hydrogels.

	Properties	Representative Materials	Mechanism of Conductivity Enhancement	Advantages	Limitations	Ref.
Materials						
Metallic Nanomaterials		Silver nanowires	Percolated metallic networks for electronic conduction	High conductivity, facile synthesis	Potential cytotoxicity, poor flexibility at high loadings	[80,81]
		Liquid metals	Flowable metallic phases with deformability	Intrinsic softness, good mechanical compliance	Stability issues, oxidation	[82,83]
		MXenes	2D layered structure allowing both ionic and electronic conduction	High surface area, excellent mixed conductivity	Sensitive to oxidation, dispersion stability	[84,85]
Carbon-based Nanomaterials		Carbon nanotubes (CNTs)	Percolated conductive networks	High conductivity, mechanical reinforcement	Aggregation, surface functionalization often required	[86,87]
		Graphene/Graphene oxide	Planar sheets forming electron pathways	High electrical and mechanical performance	Restacking tendencies, potential biocompatibility concerns	[19,88,89]
		Carbon black	Conductive filler forming percolated paths	Cost-effective, scalable	Lower conductivity than CNTs or graphene	[90,91]

## 4. Biomedical Applications of Epicardial Patch

Recent advancements in conductive and adhesive hydrogel-based epicardial patches have enabled diverse biomedical applications in cardiac diagnostics, electrical stimulation, and regenerative therapy. The unique combination of mechanical compliance, bioadhesive properties, and electrical functionality has facilitated the development of next-generation epicardial interfaces that seamlessly integrate with dynamic myocardial tissue [92].

In this chapter, we introduce recent strategies for the deployment method for epicardial patches and discuss their applications in electrophysiological signal recording, electrical stimulation, and adjuvant cell/drug delivery with electrotherapy (Table 2).

### 4.1. Minimally Invasive Deployment on Epicardial Surfaces

**Figure 4:**
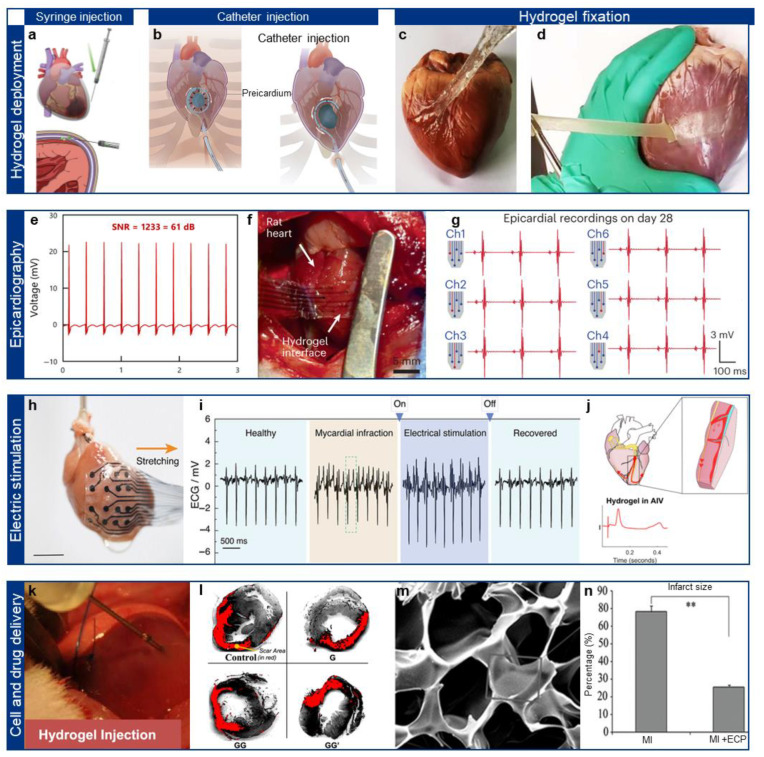
Biomedical applications of the epicardial hydrogel patch. (**a**) Schematic illustration of pericardial syringe injection of hydrogels. Reprinted with permission [97]. Copyright 2022 © Elsevier. (**b**) Catheter injection of hydrogel to the pericardial region. Reprinted with permission [98]. Copyright 2017 © Elsevier. (**c**) Viscoelastic adhesive epicardial patch. Reprinted with permission [99]. Copyright 2019 © Springer Nature. (**d**) Adhesive janus hydrogel patch for cardiac repair. Reprinted with permission [100]. Copyright 2022 © Springer Nature. (**e**) Electrocardiogram recorded with epicardial hydrogel. Reprinted with permission [58]. Copyright 2023 © Springer Nature. (**f**) Epicardial hydrogel implanted on rat heart. (**g**) Spatial recording of electrogram using epicardial hydrogel. Reprinted with permission [102]. Copyright 2023 © Springer Nature. (**h**) Epicardial adhesive hydrogel on extracted heart. (**i**) Epicardial electrogram recorded in various pathological conditions. Reprinted with permission [103]. Copyright 2024 © John Wiley and Sons. (**j**) Injectable hydrogels in coronary vein. Reprinted with permission [104]. Copyright 2024 © Springer Nature. (**k**) Injection of graphene oxide/hydrogels on heart. (**l**) Cardiac repair after gene delivery. Reprinted with permission [105]. Copyright 2014 © American Chemical Society. (**m**) SEM image of the conductive cryogel for cardiac repair. (**n**) Infarction size reduced after tissue repairing. Asterisks (**) indicate statistical significance at *p* < 0.01. Reprinted with permission [106]. Copyright 2016 © John Wiley and Sons.

Despite the growing interest in epicardial bioelectronics, the clinical translation of such devices remains challenging due to the difficulty of implantation and long-term adhesion. Traditional epicardial patches often require open-chest surgery, which increases the risk of postoperative complications and prolonged recovery time. To overcome these limitations, recent efforts have focused on developing minimally invasive strategies for delivering and securing epicardial patches, by exploiting injectable hydrogels, in situ-gelling hydrogels, and bioadhesive mechanisms [20]. Hydrogels with injectability allow for syringe or catheter-based epicardial delivery, eliminating the need for invasive procedures [54,93]. Such injectable hydrogels utilized in epicardial applications can be derived from natural sources like gelatin, hyaluronic acid, and alginate, or synthetic sources such as PEG-based hydrogels. Ideal modulus values for injectable hydrogels used in minimally invasive administration typically range from approximately 1 to 50 kPa, allowing them to maintain mechanical compatibility with cardiac tissue while facilitating easy injection. These hydrogel precursors exist as a liquid state during injection and subsequently undergo gelation in response to physiological stimuli such as temperature [94], pH [95], or enzymatic activity [96]. For instance, Li et al. developed an intrapericardial hydrogel injection strategy to enhance mesenchymal stem cell retention and therapeutic efficacy in myocardial infarction (Figure 4a) [97]. An extracellular matrix hydrogel encapsulated MSCs, improving retention (42.5 ± 7.4%) compared to intramyocardial injection (4.4 ± 1.3%). This approach improved cardiac function, reduced apoptosis, and enhanced vascular regeneration, demonstrating potential for cardiac repair. As an example for catheter-based applications, Garcia et al. developed a minimally invasive hydrogel delivery system for cardiac applications via the pericardial space (Figure 4b) [98]. A polyethylene glycol hydrogel was injected using a catheter-based device, forming a localized hydrogel compartment without systemic leakage. The method maintained stable hemodynamics, avoided arrhythmias, and demonstrated potential for epicardial therapy in cardiovascular diseases.

In addition to minimally invasive delivery, long-term adhesion and mechanical stability are crucial for the sustained functionality of epicardial patches. Bioadhesive hydrogels inspired by mussel adhesion (catechol chemistry), Schiff base bonding, and covalent crosslinking have demonstrated strong adhesion to moist cardiac tissue, even under continuous dynamic motion. Moreover, microscale interlocking structures and tissue-penetrating microstructures have been introduced to improve mechanical interfacial stability, further preventing patch detachment over time. For example, Lin et al. developed a viscoelastic adhesive hydrogel patch for epicardial application in myocardial infarction [99]. The ionically crosslinked starch hydrogel exhibited balanced fluid-solid properties (G″/G′ ~1) and strong adhesion (~60 J/m^2^). The patch adhered stably without sutures, significantly reducing left ventricular dilation and improving cardiac function in rat models (Figure 4c). In another example, He et al. developed a Janus adhesive hydrogel for minimally invasive cardiac repair and adhesion prevention [100]. The hydrogel’s conductive and viscoelastic layer adheres to the myocardium (~223.14 kPa) while the anti-adhesion layer prevents tissue synechia (Figure 4d). It enables myocardial infarction repair by reducing oxidative stress, reconstructing electrical conduction, and promoting vascularization without secondary damage.

### 4.2. Epicardial Patches for Epicardiographic Recording

Real-time monitoring of electrophysiological signals is essential for diagnosing and treating cardiac arrhythmias, ischemic events, and conduction disorders. Conductive epicardial patches could perform high-resolution mapping of epicardial electrical activity, offering several advantages over conventional endocardial catheter-based approaches [101]. For instance, Chong et al. developed a highly conductive hydrogel for cardiac signal recording using a template-directed assembly of PEDOT:PSS (Figure 4e) [58]. The hydrogel exhibited a conductivity of 247 S/cm, tissue-like modulus (25 kPa), high stretchability (610%), and tough adhesion (800 J/m^2^). Due to such optimized performance, the hydrogel enabled stable and high-quality epicardial ECG recording with an SNR of 60.08 dB in vivo.

Recent studies have also explored multiplexed and stretchable electrode designs, which allow for distributed sensing across large epicardial regions. These systems employ conductive polymer composites and liquid metal-based electrodes embedded within ultrasoft hydrogel matrices, ensuring stable signal acquisition even under continuous cardiac motion. For instance, Zhou et al. developed a 3D-printed conducting polymer hydrogel (BC-CPH) for multichannel cardiac signal recording [102]. The PEDOT:PSS-based hydrogel achieved high conductivity (>11 S/cm), stretchability (>400%), and fracture toughness (>3300 J/m^2^) (Figure 4f). Such hydrogels are patterned and fabricated into an all-hydrogel electrode array, and the integrated platform enabled stable and high-fidelity epicardial ECG recording (SNR > 60 dB) over 28 days in rat models (Figure 4g).

### 4.3. Epicardial Patches for Epicardial Stimulation

Epicardial patches have been widely explored for therapeutic electrical stimulation, offering a promising alternative to implantable pacemakers and defibrillators. Conductive hydrogel-based patches allow for localized and tunable electrical stimulation, reducing the risk of off-target tissue activation. Electrically conductive hydrogels composed of PEDOT:PSS or silver nanowire (Ag NW) composites provide a low-impedance interface, enabling efficient current delivery to epicardial tissue. Soft hydrogel pacemakers have been developed to synchronize with natural cardiac rhythms, reducing mechanical stress and foreign body reactions compared to rigid pacing electrodes. Recent studies have demonstrated that hydrogel-based epicardial stimulators can precisely modulate cardiac conduction, showing promise for personalized cardiac therapy. For instance, Wang et al. developed a 3D-printed PEDOT:PSS hydrogel bioelectronic device for cardiac signal monitoring and electrical stimulation [103]. The hydrogel exhibited a conductivity of >9 S/m, Young’s modulus of 650 kPa, and interfacial toughness of 200 J/m^2^, ensuring stable bioadhesion (Figure 4h). It enabled high-fidelity epicardial ECG recording (SNR ~ 50) and effective electrical stimulation (50 mV, 8 Hz, 4 ms) to restore cardiac rhythm in infarcted rat hearts (Figure 4i). In other example, Rodriguez-Rivera et al. developed an injectable hydrogel electrode for minimally invasive cardiac pacing and conduction restoration [104]. The PEG-based ionic hydrogel (conductivity: 13.5 ± 0.8 mS/cm) was injected into coronary veins, forming conduction highways that captured mid-myocardial tissue and improved pacing efficiency (Figure 4j). In porcine models, hydrogel pacing achieved QRS morphology similar to native sinus rhythm and normalized conduction delays in infarcted myocardium, demonstrating potential for treating ventricular arrhythmias and enabling painless defibrillation.

### 4.4. Epicardial Patches for Drug and Cell Delivery

Discussing hydrogel property for application in drug and cell delivery, which can compensate limitations of conventional therapies, would be beneficial for the epicardial hydrogel electronics [107,108]. Cocktail therapy including electrotherapy, chemotherapy, and cell therapy would be a great advantage in terms of therapeutic efficacy, considering their synergistic effect. Recent advances in targeting and localized delivery strategies, including stimulus-responsive hydrogels and nanoparticle-functionalized systems, have significantly improved the specificity and efficacy of therapeutic interventions provided by epicardial patches. Targeting delivery could enhance the delivery efficacy to target cells and minimize the side effects to normal cells in a microscopic environment, while localized delivery could enhance the delivery efficacy to target tissue and minimize the side effects to other tissues in a macroscopic environment. They synergistically result in effective, precise, and controlled therapeutic outcomes.

Controlled drug delivery systems integrated into epicardial patches can release anti-inflammatory agents (e.g., dexamethasone) [109], angiogenic growth factors (e.g., VEGF) [110], or regenerative molecules (e.g., IGF-1) [111] over prolonged periods. The release behavior of such materials is highly dependent on the drug reservoir types, such as diffusion-based hydrogels, electro-responsive hydrogels, and nanoparticle-loaded hydrogels. Specifically, electrically responsive hydrogels can be integrated with drug delivery systems to implement on-demand drug release, triggered by external electrical stimulation, enhancing therapeutic control. For example, Paul et al. developed an injectable graphene oxide (GO)-functionalized hydrogel for localized gene delivery in myocardial infarction therapy [105]. A methacrylated gelatin (GelMA) hydrogel encapsulated PEI-functionalized GO carrying VEGF DNA, enabling sustained VEGF release (Figure 4k). The system enhanced capillary density (148.7 ± 21.2/mm^2^), reduced the scar area (19.4 ± 5.8%), and improved cardiac function (ejection fraction: 49.4 ± 3.8%) in infarcted rat hearts (Figure 4l).

Stem cell-laden hydrogel patches (e.g., iPSC-derived cardiomyocyte sheets) have demonstrated improved cell survival and functional integration with host myocardium. Conductive hydrogel scaffolds provide electrical coupling between transplanted cells and native tissue, promoting synchronized excitation-contraction activity. For instance, Wang et al. developed a mussel-inspired conductive hydrogel for cardiac cell delivery and infarct repair [106]. A polypyrrole (Ppy)-gelatin-PEG cryogel with dopamine crosslinking exhibited high elasticity (Young’s modulus ~300 kPa) and conductivity (0.0072 S/m) (Figure 4m). Cardiomyocyte-loaded patches improved myocardial retention, reducing infarct size by 42.6% and enhancing ejection fraction by ~50% in rat MI models (Figure 4n). These advances highlight the potential of epicardial hydrogel patches for cardiac repair, combining electrical stimulation with drug and cell-based therapies (Table 3).

## 5. Current Limitations and Prospects

Despite significant advancements in the development of epicardial hydrogel patches, several critical challenges hinder their widespread clinical translation. One of the most pressing issues is mechanical and adhesive stability. The heart is a continuously contracting and relaxing organ, subjecting any implanted material to constant shear and tensile forces. While bioadhesive hydrogels have demonstrated strong initial adhesion through mechanisms such as covalent bonding (Schiff base reactions), electrostatic interactions, and catechol-inspired adhesion, many fail to maintain stable long-term attachment. Over time, hydration effects and mechanical fatigue can lead to delamination or displacement of the hydrogel patch, reducing its effectiveness as an electrical or therapeutic interface. The softness of hydrogels, which is beneficial for minimizing mechanical mismatch, can also render them structurally fragile, especially under prolonged cyclic loading. To address these limitations about long-term stable fixation, future research should focus on developing dynamic covalent bonding strategies that provide reversible yet strong adhesion, while exploring mechanically interlocking architectures that physically integrate with the epicardium without excessive reliance on chemical modification (Table 4).

Another major challenge lies in the electrical conductivity and functional longevity of hydrogel-based patches. For applications such as epicardial conduction mapping and electrical stimulation, maintaining stable conductivity over time is crucial. Most conductive hydrogels rely on ionic charge transport, achieved through the incorporation of electrolytes or ionic liquids, but these materials often suffer from ion leakage, which gradually depletes conductivity and affects the surrounding ionic balance. Conductive polymer-based hydrogels, such as PEDOT:PSS composites, provide electronic conductivity but can experience phase separation, polymer degradation, or mechanical stiffening over time, reducing their long-term stability. To mitigate these issues, researchers are exploring nanocomposite strategies, integrating highly stable conductive nanomaterials, such as MXenes, carbon nanotubes, or graphene hybrids, to sustain conductivity while enhancing mechanical durability. Additionally, the development of self-healing conductive hydrogels that restore electrical connectivity after mechanical deformation could significantly improve patch longevity and performance.

Beyond mechanical and electrical considerations, potential immune responses such as fibrotic encapsulation and foreign body reactions remain significant concerns for long-term hydrogel implantation. Although hydrogels are generally regarded as biocompatible, protein adsorption and subsequent foreign body responses can occur, leading to fibrotic encapsulation and isolating the patch from cardiac tissue and impairing its function. Moreover, degradation products of the hydrogel can trigger inflammatory reactions, further exacerbating tissue responses. Strategies to minimize these effects include engineering hydrogel coatings with antifouling properties, such as zwitterionic or hyaluronic acid-based polymers, which have been suggested to reduce protein adsorption and subsequent immune activation. Incorporating immunomodulatory agents, such as IL-10-releasing nanoparticles, may further help suppress chronic inflammation and enhance long-term biocompatibility.

Another challenge that must be addressed for clinical translation is integrability, which emphasizes the possibility of integration for epicardial hydrogel patches with existing cardiac medical devices to implement closed-loop systems. Most hydrogel patches developed in research settings lack wireless communication capabilities or real-time adaptive control, making them less practical for real-world applications. Conventional implantable cardiac devices, such as pacemakers and defibrillators, rely on rigid electronic components that are difficult to interface with soft and deformable hydrogel structures. Bridging this gap requires cutting-edge technology about bioelectronic integration, such as wireless energy harvesting technologies (e.g., near-field communication or biofuel cells) that eliminate the need for external power sources, and machine-learning-driven closed-loop control systems that enable patches to autonomously adjust stimulation parameters based on real-time cardiac signals [112]. Moreover, recent advancements in integrating epicardial patches with wireless and implantable electronics have shown great potential for enabling continuous, real-time data transmission. Technologies such as near-field communication, Bluetooth, and radio-frequency identification have been integrated, significantly enhancing the functionality of these patches by enabling remote monitoring and dynamic therapeutic adjustments.

Additionally, improving scalability and reproducibility in hydrogel fabrication, through techniques such as 3D bioprinting or microfluidic templating, will be essential for translating these technologies from laboratory prototypes to clinically viable products.

Looking forward, the next generation of epicardial hydrogel patches is expected to integrate multiple functionalities, combining bioadhesion, conductivity, mechanical compliance, and therapeutic capabilities into a monolithic platform. Advances in synthetic biology, smart biomaterials, and nanotechnology may render hydrogel patches to dynamically respond to physiological cues, adapting their properties in real-time for optimizing therapeutic outcomes. While preclinical studies in small and large animal models have demonstrated promising results, further validation through long-term implantation studies and human clinical trials will be necessary to fully establish the safety, efficacy, and reliability of these devices.

Despite these challenges, epicardial hydrogel patches hold great potential to revolutionize cardiac care, providing minimally invasive, biocompatible, and multifunctional solutions for continuous electrophysiological monitoring, electrical stimulation, and targeted cardiac therapies. With continued research and interdisciplinary collaboration, these technologies may pave the way for the next generation of implantable cardiac bioelectronics, offering personalized and adaptive treatments for patients with heart disease.

## Figures and Tables

**Table 2:** Systematic comparison of epicardial patch materials and designs for epicardiographic recording.

Patch Material	Conductivity (S/cm)	Modulus (kPa)	Stretchability (%)	Signal-to-Noise Ratio (dB)
Electronically conductive hydrogel	>10	25–100	400–610	>60
Nanocomposite hydrogel	~20	50–150	~300	~55
Ionic conductive hydrogel	5–10	10–50	>500	40–50

**Table 3:** Comparative analysis of hydrogel systems for drug versus cell delivery.

Hydrogel Type	Typical Composition	Primary Application	Controlled Release Mechanism	Key Advantages
Drug Delivery Hydrogel	PEG, GelMA, alginate	Anti-inflammatory, angiogenic factor delivery	Diffusion-based, stimuli-responsive release	Precise dosage, sustained release
Cell Delivery Hydrogel	Gelatin, hyaluronic acid, fibrin	Stem cell, cardiomyocyte encapsulation	Cell encapsulation, mechanical protection	Enhanced cell viability, tissue integration

**Table 4:** Representative binding strategies between epicardial tissue and hydrogels.

Binding Strategy	Mechanism	Key Features
Schiff Base	Aldehyde-amine reaction	Reversible, stable under physiological conditions
Boronate Ester	Diol-boronic acid reaction	pH-responsive reversibility
Diels–Alder	Diene-dienophile cycloaddition	Temperature-responsive reversibility
Host–Guest Interaction	Supramolecular cyclodextrin-adamantane binding	Highly reversible, rapid self-healing
Catechol-based	Mussel-inspired dopamine adhesion	Strong adhesion, redox-mediated reversibility

## Data Availability

No new data were created or analyzed in this study.
